# Ligand Activation of PPARγ by Ligustrazine Suppresses Pericyte Functions of Hepatic Stellate Cells via SMRT-Mediated Transrepression of HIF-1α: Erratum

**DOI:** 10.7150/thno.73098

**Published:** 2022-04-26

**Authors:** Feng Zhang, Shuai Lu, Jianlin He, Huanhuan Jin, Feixia Wang, Li Wu, Jiangjuan Shao, Anping Chen, Shizhong Zheng

**Affiliations:** 1 Jiangsu Key Laboratory for Pharmacology and Safety Evaluation of Chinese Materia Medica, Nanjing University of Chinese Medicine, Nanjing 210023, China.; 2 Third Institute of Oceanography, State Oceanic Administration, Xiamen 361005, China.; 3 Jiangsu Key Laboratory of Therapeutic Material of Chinese Medicine, Nanjing University of Chinese Medicine, Nanjing 210023, China.; 4 State Key Laboratory Cultivation Base for TCM Quality and Efficacy, Nanjing University of Chinese Medicine, Nanjing 210023, China.; 5 School of Science, China Pharmaceutical University, Nanjing 211198, China.; 6 Department of Pathology, School of Medicine, Saint Louis University, MO 63104, USA.

The authors regret that the original version of this paper unfortunately contained an inappropriate representative image of Boyden chamber assay. An inappropriate image was inadvertently used for the HSC-LX2 Ligu+GW9662 (Figure 4F) during the assembly of this figure. The authors confirm that the correction of Figure 4F does not affect the original conclusions. The authors sincerely apologize for any inconvenience or misunderstanding that the error may have caused. The corrected image is shown below.

## Figures and Tables

**Figure 1:**
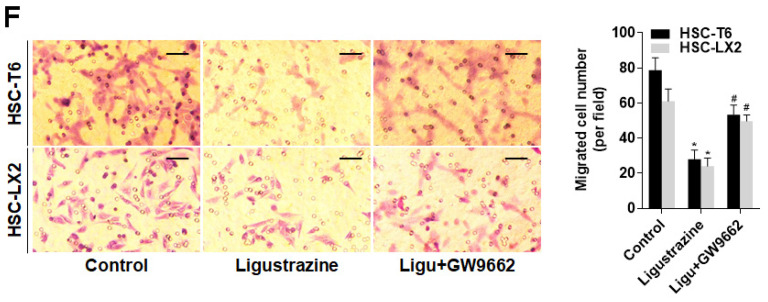
Corrected image for original Figure 4F.

